# The probability and severity of decompression sickness

**DOI:** 10.1371/journal.pone.0172665

**Published:** 2017-03-15

**Authors:** Laurens E. Howle, Paul W. Weber, Ethan A. Hada, Richard D. Vann, Petar J. Denoble

**Affiliations:** 1 Department of Mechanical Engineering and Materials Science, Hudson Hall, Research Drive, Duke University, Durham, NC United States of America; 2 Department of Radiology, Duke University Medical Center, Durham, NC United States of America; 3 BelleQuant Engineering, PLLC, Mebane, NC United States of America; 4 Divers Alert Network, 6 West Colony Place, Durham, NC United States of America; Vanderbilt University Medical Center, UNITED STATES

## Abstract

Decompression sickness (DCS), which is caused by inert gas bubbles in tissues, is an injury of concern for scuba divers, compressed air workers, astronauts, and aviators. Case reports for 3322 air and N_2_-O_2_ dives, resulting in 190 DCS events, were retrospectively analyzed and the outcomes were scored as (1) serious neurological, (2) cardiopulmonary, (3) mild neurological, (4) pain, (5) lymphatic or skin, and (6) constitutional or nonspecific manifestations. Following standard U.S. Navy medical definitions, the data were grouped into mild—Type I (manifestations 4–6)–and serious–Type II (manifestations 1–3). Additionally, we considered an alternative grouping of mild–Type A (manifestations 3–6)–and serious–Type B (manifestations 1 and 2). The current U.S. Navy guidance allows for a 2% probability of mild DCS and a 0.1% probability of serious DCS. We developed a hierarchical trinomial (3-state) probabilistic DCS model that simultaneously predicts the probability of mild and serious DCS given a dive exposure. Both the Type I/II and Type A/B discriminations of mild and serious DCS resulted in a highly significant (p << 0.01) improvement in trinomial model fit over the binomial (2-state) model. With the Type I/II definition, we found that the predicted probability of ‘mild’ DCS resulted in a longer allowable bottom time for the same 2% limit. However, for the 0.1% serious DCS limit, we found a vastly decreased allowable bottom dive time for all dive depths. If the Type A/B scoring was assigned to outcome severity, the no decompression limits (NDL) for air dives were still controlled by the acceptable serious DCS risk limit rather than the acceptable mild DCS risk limit. However, in this case, longer NDL limits were allowed than with the Type I/II scoring. The trinomial model mild and serious probabilities agree reasonably well with the current air NDL only with the Type A/B scoring and when 0.2% risk of serious DCS is allowed.

## Introduction

Decompression sickness (DCS) is an injury that is a concern for scuba divers, compressed air workers, astronauts, aviators and other personnel exposed to hyperbaric and/or hypobaric environments. Although DCS can result from both hyper- and hypobaric exposures, we focus only on hyperbaric exposures for the present work. The principal cause of DCS is supersaturation of inert gas in the bloodstream and tissues leading to the formation of gas bubbles [1]. Symptoms of DCS can range from relatively harmless manifestations such as slight pains in the joints to devastating symptoms such as paralysis and death. The evolution (especially as it relates to hyper/hypobaric exposure) and treatment of DCS are still active areas of research.

Models which attempt to mitigate the occurrence of DCS have been in use for over a century. The first known model to prescribe decompression schedules following hyperbaric exposures was the Haldane decompression algorithm, which was introduced to the diving practice at the beginning of the 20^th^ Century [2]. Haldane’s decompression algorithm drastically reduced the prevalence and severity of DCS by dictating the schedule by which a diver could safely return to the surface after a given hyperbaric exposure. Although the Haldane model reduced the instances of DCS in the field, it was not perfect, as some divers that followed “safe” recompression schedules still experienced DCS.

DCS model improvement continued throughout the 20^th^ Century and still continues today. DCS models may be broken into two main categories, deterministic and probabilistic. Deterministic models (such as the Haldane model) are binary in outcome and predict whether or not a diver will experience DCS after a given dive profile. Deterministic models leave no middle ground when it comes to predicting DCS; the model prediction is that the diver will or will not experience DCS. This is a problem because empirical data has shown that DCS may occur for only a small percentage of many divers that all execute the exact same dive. Additionally, the symptoms for the divers that experience DCS after executing the exact same dive may vary; drastically in some cases [3–6].

Probabilistic DCS modeling, first introduced into the field by Weathersby *et al*. [4], assumes a non-zero probability of experiencing DCS for a given dive. Probabilistic modeling is advantageous because the parameters of the model may be calibrated from empirical data, and different models may be compared (formally or informally) based on statistical tests. Decompression models have evolved to the point that differences between decompression algorithms can no longer be distinguished by casual observation. Differences can be distinguished, however, by DCS probability estimation if adequate dive data are available [4].

DCS models in use today have the drawback that although they predict the occurrence and/or probability of DCS, they do not predict the severity of the DCS manifestations. Predicting the severity of DCS for a given dive would be advantageous because it would allow for safety analysis to be conducted. Safety is defined as freedom from the risk of injury [7, 8]. However, complete safety cannot always be guaranteed because risk is characteristic of many human activities (including decompression), and many activities such as military diving must accept some risk.

Both the probability and severity of injury are used to determine risk, and risk increases as either the probability or severity rises [9]. A high-risk activity could have a low probability of serious injury or a high probability of mild injury. Typically, a risk assessment matrix is used in which the estimated probability appears vertically and the severity horizontally [10]. Each matrix cell combines information about probability and severity and is assigned a risk assessment code ranging from negligible to catastrophic. The U.S. Navy and Marine Corps, for example, use a risk assessment matrix for managing operational risks [11]. Assigning risk assessment codes and judging what constitutes acceptable risk are personal decisions for an individual and social or political decisions for an organization. Inherent in the definition of safety is that if the severity of injury is not known to a high level of confidence, then conservative estimates of the severity of injury must be made, such as assuming all DCS events have a high probability of serious injury, which may unnecessarily limit the level of activity.

Although no DCS models in use today predict the severity of DCS, the importance of categorizing the risk and severity of DCS and realizing its operational consequences has been previously considered in the diving community. The U.S. Navy adopted the approach that: “The tolerable risk for a given dive is a matter of policy and may vary with the circumstances. Discussions at the Naval Sea Systems Command brought consensus that more than two cases of Type I (pain-only) or minor Type II (neurological or cardiopulmonary) DCS per 100 dives in routine U.S. Navy diving would hurt diver morale and would slow operational tempo; for serious neurological or cardiopulmonary DCS, the maximum acceptable incidence is one case per 1,000 dives (personal communication, Murray CA; 2000)” [12]. This statement makes two very important points. The first is that DCS should be categorized by severity as opposed to a simple binary (yes/no) occurrence. Type I DCS, also known as ‘mild’ DCS, was defined as pain-only symptoms, whereas Type II DCS, also known as ‘serious’ DCS, was defined as neurological or cardiopulmonary symptoms. The second point is that the U.S. Navy was willing to accept a higher probability for ‘mild’ DCS (2%) than for ‘serious’ DCS (0.1%). Later in this paper, we will examine the consequences of this 20:1 ratio with regards to the USN 2008 air no decompression limit (NDL) tables.

Shields and Lee [13] also described the importance of distinguishing by severity when determining decompression safety: “In considering ‘acceptability,’ one must take into account not only the overall incidence of DCS, but also its manifestations. Pain-only limb bends, although not desirable, might be acceptable as an occupational hazard of diving; neurological DCS with the possibility of cumulative and perhaps permanent damage, is not.” An overall DCS incidence of less than 0.5% was acceptable for Shields and Lee while “the only acceptable incidence for Type II DCS in an occupational situation…is zero.” Corresponding opinions by U.S. commercial diving operators indicated that 0.02–0.1% was acceptable for Type I DCS while 0–0.025% was acceptable for Type II DCS [14].

The acceptable percentages of mild and serious DCS described by the U.S. Navy and others may apply to either the actual incidences of mild and serious DCS during unrestricted diving operations or to the maximum probabilities of mild and serious DCS predicted by a decompression model for a specific dive profile. In either case, exceeding the acceptable incidences or probabilities would be undesirable. If separate acceptable risks are to pertain to mild and serious DCS, probabilistic decompression models must be calibrated accordingly and appropriate diving procedures selected. This can be accomplished by applying multinomial probability models to empirical calibration data as opposed to the binomial probability models in use today. “Multinomial” means that more than one probability is predicted simultaneously; for example, a trinomial model may simultaneously predict the probabilities of mild DCS, serious DCS and no DCS.

Deciding what constitutes mild and serious DCS and how they are to be measured are greater challenges. When a diving physician assigns a diagnosis of DCS to a diver, serious manifestations (usually designated as "Type II”) take precedence over mild manifestations (usually designated as “Type I”). If an individual should have both Type I and II manifestations, the case is reported as Type II. We refer to this as a “hierarchical” classification. To determine if Type I manifestations were also present, it is necessary to consult the clinical case descriptions that list all manifestations found during clinical examination. With this information, the probabilities of mild (*P*_*m*_) and serious (*P*_*s*_) DCS can be estimated. We refer to *P*_*m*_ and *P*_*s*_ as “competitive” rather than as hierarchical probabilities.

In the present work, we explore two definitions of mild and serious DCS that were based upon clinical judgment. We have also previously investigated a method for discriminating between definitions of DCS severity based on survival analysis [15]. Ultimately, these are judgments that must be made by the developers and users of the decompression procedures, as each organization will define different levels of acceptable risk according to their operational requirements.

One approach in developing a DCS modeling system that predicts the probability of DCS occurrence for events with differing severity would be to separately fit models to the different DCS types present in the calibration data. The attraction of this approach is that different physiological models could be used for different severity types. A drawback of this approach is that event types with sparse representation in the calibration data set (for example, cardiopulmonary manifestations) might not possess a sufficient number of events to justify the number of free parameters in the model, resulting in over-fitting. In addition, this approach does not allow for the interplay between the probabilities (i.e., competitive vs. hierarchical) of different DCS types which results from measurement bias.

For the present work, we take a simpler approach to the problem of modeling DCS severity by developing a framework that can be applied to many DCS models already in existence. This will allow for the model to be fit to DCS events of differing severity simultaneously. The attraction of this approach is that only a single additional free parameter is needed for each additional type of DCS severity. In constructing this framework, it is necessary to develop the connection between the model’s competing (predicted) and hierarchical (observed) probabilities. While this approach does not, in the present work, allow for the model dynamics to vary between the events of different severity, it does allow for the fitting to rare DCS events and allows for a rigorous study of fit improvement with the added parameters.

## Methods

In the Methods subsections to follow, we discuss the data set used for model calibration, present our rationale for discriminating the event severity, briefly discuss the DCS model used for this work, and develop our trinomial (3-state) model. This is followed by a derivation of the link between the model competing and hierarchical probabilities, presentation of the multinomial likelihood function used to fit the probabilistic DCS model to the empirical dive data, and a derivation of equivalent likelihoods. For optimizing the DCS model, we derive exact expressions for model gain and scaling parameters so that these parameters are removed from the optimization space. Our final two Methods subsections discuss the model optimization system and the statistical methods used for this work.

### Calibration data set

For all of the model fitting in this work, we used the BIG292 standard DCS data set available in two NMRI reports [5, 6]. This data set contains the dive profiles and diver outcome information (i.e., whether or not DCS occurred) for 3322 Air and N_2_-O_2_ exposures that cover depths ranging from 20 to 602.4 feet of seawater (fsw) and durations ranging from 0.64 to 12,960 min (including surface intervals for repetitive dives). The dive trials included in this data set were conducted by the U.S., U.K., and Canadian militaries between 1944 and 1997. The data set contains 190 cases of decompression sickness, and 110 cases of marginal decompression sickness. Outcomes were considered marginal (or “niggles”) when symptoms were mild, short duration (<60 min for 1 joint, <30 min for multiple joints) aches, pains, or fatigue that spontaneously resolved without recompression treatment [5, 6]. The use of marginal DCS events in the fitting of probabilistic DCS models is discussed in detail elsewhere [16].

Symptom case histories for the full and marginal DCS cases are presented in the reports along with symptom onset times for all of the DCS cases and for 68/110 of the marginal DCS cases. The symptom onset times are given as *T*_1_, the last time the diver was known to definitely be asymptomatic, and *T*_2_, the first time the diver was definitely known to be symptomatic. For all of the models in this paper, we used the symptom onset times when calculating the probabilities of suffering DCS as detailed elsewhere [17]. As uncertainty could be associated with the determination of T_1_ and T_2_, rules were established so they could be assigned consistently [18], but assigned onset times might be minutes to hours different from the actual onset time. Because all of the data used in this study are anonymized and de-identified and are available to the public without restriction in two official Government reports, no IRB approval was required for the present work.

### Discrimination by DCS event severity

Descriptions of the DCS cases and marginal events were published in the NMRI reports so investigators could make independent judgments of diagnoses [5, 6]. We transformed the symptom descriptions into a numerical value using the empirical six category scale shown in Table 1. We refer to this scale as the “Perceived Severity Index” (PSI) as the categories are in the order of what is generally perceived to be of decreasing severity [19, 20]. Marginal incidents (niggles) were not assigned a PSI. The PSI indices have the following definitions:

1. Serious Neurological: dysfunction involving bladder, bowel, gait, or coordination (ataxia), reflexes, mental status (dysphasia, mood, memory, orientation, personality), vision, hearing (tinnitus), consciousness, strength, vertigo.
2. Cardiopulmonary: cough, hemoptysis, dyspnea, voice change.
3. Mild Neurological: paresthesia, numbness, tingling, altered sensation.
4. Pain: ache, cramps, discomfort, joint pain, pressure, spasm, stiffness.
5. Lymphatic or Skin: edema, itching, rash, burning sensation, marbling.
6. Constitutional or Nonspecific: dizziness, fatigue, headache, nausea, vomiting, chills, diaphoresis, malaise, restlessness.

**Table 1:** Distribution of Perceived Severity Index (PSI) in the BIG292 data set with corresponding Type I/II and Type A/B classifications [19, 20].

PSI	Manifestation	Occurrences	Type I/II	Type A/B
1	Serious neurological	18	38 Type II	20 Type B
2	Cardiopulmonary	2		
3	Mild neurological	18		170 Type A
4	Pain	150	152 Type I	
5	Skin	1		
6	Constitutional	1		
	Total	190	190	190

Several PSI categories could be assigned to a given DCS case, but as the categories were hierarchical, cases could also be described by the category of greatest severity. Thus, a case with serious neurological manifestations (PSI = 1) could also have pain (PSI = 4) but would be assigned a hierarchical category of PSI = 1. Conversely, a case categorized as PSI = 4 due to pain could not have serious neurological manifestations. The PSI system collapses into the traditional DCS severity categories of Type I (PSI = 4–6) for ‘mild’ and Type II (PSI = 1–3) for ‘serious’ as indicated in Table 1. When applied to the 190 DCS cases in BIG292, 152 were of Type I and 38 were of Type II. The Type I & II classification of DCS severity is not the only classification system currently in use. In the workshop *Describing Decompression Illness*, Francis and Smith reported that, “It was agreed that patients who had only sensory changes had sustained a less severe injury than those in which there is also motor involvement” [21]. Similarly, the workshop on the *Management of Mild or Marginal Decompression Illness in Remote Locations* offered the following definition of “mild” symptoms and signs [22]:

- Limb pain where: (a) the severity of pain has little prognostic significance, but may influence management decisions independent of the classification of pain as a “mild” symptom; and (b) classical girdle pain syndromes are suggestive of spinal involvement and do not fall under the classification of “limb pain.”
- Some cutaneous sensory changes that include subjective cutaneous sensory phenomena such as paraesthesiae that are present in patchy or non-dermatomal distributions suggestive of non-spinal, non-specific, and benign processes. Subjective sensory changes in clear dermatomal distributions or in certain characteristic patterns such as in both feet, may predict evolution of spinal symptoms and should not be considered “mild.”
- Constitutional symptoms or rash
- Objective neurological dysfunction must be excluded by medical examination. The proclamation of “mild” cannot be made where symptoms are progressive. The “mild” designation must be repeatedly reviewed over at least 24 hours following diving or the most recent recompression if there was ascent to altitude.

These workshops suggest an alternative approach to DCS severity in which mild neurological symptoms (PSI = 3) would be classified as Type I rather than Type II DCS. For clarity, we refer to this classification scheme as Type A & B rather than Type I & II. BIG292 contains 170 Type A and 20 Type B DCS cases (Table 1). In the discussion below, we treat DCS severity as ‘Mild’ or ‘Serious’ where these are defined as in Table 1 by Type I or Type II and by Type A or Type B. To develop our concepts and procedures, most of our work defined ‘mild’ as Type A DCS and ‘serious’ as Type B DCS. However, because Type I & II definitions are in common use, we also explored the consequences of defining Type I as mild and Type II as serious. As we will demonstrate in the results section, the classification of severity by Types I/II or Types A/B has important implications for operational diving exposure limits.

### DCS model

Although the methodology we present for constructing hierarchical probabilities is general and can be applied to many existing probabilistic decompression models, we chose the LE1nt model for this work [23, 24]. This model employs three parallel, perfusion-limited compartments, each with a unique half-time, and allows for a switch between exponential and linear gas kinetics in the intermediate compartment. The “nt” designation indicates that this particular model does not use a threshold term in the slow compartment as do some variants of this model class. A detailed derivation of this model and the exact solution for the exponential-to-linear gas kinetics cross-over conditions is available in our previous work [25]. In addition to our having experience with this model, it serves as the basis for several other DCS models, such as the NMRI98 model [26]. The particular variant of LE1nt that we use for this work has three parallel, well-perfused tissue compartments, does not use pressure thresholds in the definition of the risk function and only uses an exponential to linear cross-over pressure in the intermediate tissue compartment. Additionally, this variant of the LE1nt model contains 7 adjustable parameters (3 gains, 3 tissue time-constants, 1 cross-over pressure). The exact gains can be found implicitly and eliminated from the optimization so that only 4 adjustable parameters remain for the binomial (2-state) model [27]. The trinomial (3-state) model adds one additional adjustable parameter to the binomial model.

### Trinomial model

Weathersby *et al*. [4], Thalmann *et al*. [23], and others treated DCS as a binomial process (no DCS, DCS) with the probability of developing DCS defined as

$$P_{DCS}=1-e^{-\vec{g}•\vec{R}}$$ (1)

with the probability of not developing DCS defined as

$$P_{0}=1-P_{DCS}=e^{-\vec{g}•\vec{R}}.$$ (2)

In Eqs (1) and (2), $\vec{g}$ is a vector of gain parameters and $\vec{R}$ is a hazard vector containing the compartmental risk information. Our trinomial model definition scales the probability of serious DCS from that of mild DCS using a scale factor, *a*, so that

$$\begin{array}{l} P_{s}^{c}=1-e^{-a\left(\vec{g}•\vec{R}\right)}\\ P_{m}^{c}=1-e^{-\vec{g}•\vec{R}}. \end{array}$$ (3)

In Eq (3), the subscript indicates the mild or serious state and the superscript indicates that these expressions are competitive probabilities. The choice of which event is scaled by the additional parameter, *a*, is arbitrary. However, we expect that the convergence of the model parameters will be improved if we do not add the additional parameter to the most frequently occurring event. Therefore, we elected to add the additional parameter to the less frequently occurring serious DCS event. Note that other definitions for the competitive probabilities are possible. The definitions we use offer the advantage that one additional parameter allows the same DCS model to distinguish between mild and serious DCS. Note that this formulation does not allow for the time of symptom onset to vary between the different events, although we expect that this method could be extended to allow different dynamics for different event severities.

### Competitive and hierarchical probabilities

The DCS outcomes are graded with priority given to the most serious DCS manifestations. This reporting of a single outcome from a multinomial process removes the joint probability that a diver experiences multiple symptoms from the observations, creating a hierarchical system. For example, serious DCS takes precedence over mild DCS which takes precedence over marginal DCS. As a result, the hierarchical probability of mild DCS must be multiplied by the probability of the diver not experiencing serious DCS and so on. In order to write the hierarchical probabilities compactly, we will use the notation

$$e^{-\vec{g}•\vec{R}}=\xi$$ (4)

so that

$$e^{-a\left(\vec{g}•\vec{R}\right)}=\xi^{a}$$ (5)

and the mathematics of exponents and logarithms apply as usual. The hierarchical probabilities, in terms of the competitive probabilities, are then

$$\begin{array}{l} P_{s}^{h}=P_{s}^{c}=1-\xi^{a}\\ P_{m}^{h}=P_{m}^{c}\left(1-P_{s}^{h}\right)=\xi^{a}-\xi^{a+1} \end{array}$$ (6)

The probability of the non-event state for the trinomial models is

$$P_{0,tri}^{h}=1-P_{s}^{h}-P_{m}^{h}=\xi^{a+1}$$ (7)

For both the binomial and trinomial models, the sum of the probabilities of the events and the non-events equals 1, as is required by the law of total probability.

**Fig 1:**
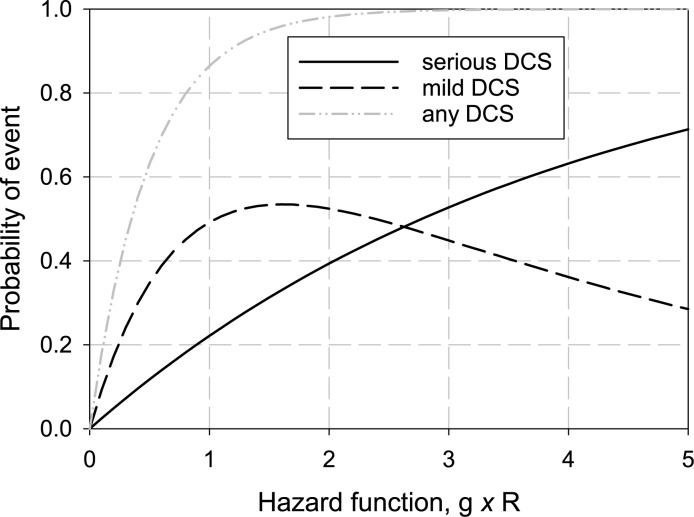
Probabilities of DCS events of differing severity with increasing value of the hazard function in the hierarchical model. Because serious DCS masks mild DCS, as the probability of serious DCS increases, the probability of observing mild DCS decreases. A scale factor of *a* = 0.25 was used to generate these results.

The distinction between the competitive and hierarchical probabilities is subtle but important. The hierarchical probabilities are reported (observed) and the competitive probabilities are computed by Eq (3). A plot of the hierarchical probabilities as a function of the hazard function for a single tissue is shown in Fig 1 for *a* = 0.25. This value of scale parameter is reasonably realistic for the Type I/II splitting of mild and serious events. As the figure indicates, all event probabilities first increase. Then, the hierarchical probability of mild DCS diminishes as the probability of serious DCS increases making it less likely for a diver to suffer mild DCS only.

### Multinomial likelihood functions

The adjustable parameters for the binomial and trinomial models are optimized through the method of log likelihood maximization [4], although the statements of log likelihood are different for the different models. For the binomial problem, the log likelihood is

$$LL_{2}=\sum_{i=1}^{N}\ln\left[{\left(1-P_{D,i}\right)}^{1-\delta }P_{D,i}{}^{\delta }\right]$$ (8)

where *P*_*D*,*i*_ is the probability of DCS on dive profile *i* of the data set and *δ* = 1 if the dive resulted in DCS and *δ* = 0 otherwise. We do not consider fractional weighting of marginal DCS events for either the binomial or trinomial models. This function has been examined in detail in the context of probabilistic modeling of DCS elsewhere [15]. For the trinomial model, the appropriate log likelihood expression is

$$LL_{3}=\sum_{i=1}^{N}\ln\left[{\left(1-P_{m,i}^{h}-P_{s,i}^{h}\right)}^{1-\mu -\sigma }{\left(P_{m,i}^{h}\right)}^{\mu }{\left(P_{s,i}^{h}\right)}^{\sigma }\right]$$ (9)

where *μ* = *σ* = 0 for no DCS, *μ* = 1, *σ* = 0 for mild DCS and *μ* = 0, *σ* = 1 for serious DCS. As a result of our previous study on the use of marginal events in fitting DCS models [16], we group the marginal DCS profiles with the non-event profiles. We will return to these expressions during our derivation of optimal gain and scaling parameters.

### Equivalent log likelihoods

In order to compare the relative performance of nested models, the log likelihood difference test is useful [28]. We would like to design a test to see whether adding an additional adjustable parameter to step from the binomial to the trinomial model is justified by an appropriate change in log likelihood. A direct comparison between *LL*_2_ and *LL*_3_ is meaningless because the data are graded differently. However, we can deflate *LL*_3_ to generate the equivalent *LL*_2_ via:

$$LL_{32}=\sum_{i=1}^{N}\ln\left[{\left(1-P_{m,i}^{h}-P_{s,i}^{h}\right)}^{1-\mu -\sigma }{\left(P_{m,i}^{h}+P_{s,i}^{h}\right)}^{\mu +\sigma }\right]$$ (10)

In this last expression, the subscripts *LL*_*xy*_ denote the *x* state model deflated to the *y* state problem and the bit-field rules of Eq (9) still apply. Now, Eq (10) can be directly compared to Eq (8) for log likelihood difference hypotheses testing.

We will now construct a simple example that illustrates the role of competitive probabilities, hierarchical probabilities, and deflated (equivalent) log likelihoods. Consider a simple process that selects a pair of independent random numbers, each with uniform probability, on a unit interval. If the first random number is less than 0.2, event A occurs and if the second random number is less than 0.2, event B occurs. However, the first number is observed before the second, and if the first is found to be less than 0.2, event A is recorded and that trial halts. This adds a bias into the observation by masking the joint probability of events A and B (*P*_*A*_*P*_*B*_) from being recorded. The conditional probabilities of events A and B are $P_{A}^{c}=P_{B}^{c}=0.2$. The hierarchical (biased, recorded) probabilities of events A and B are

$$\begin{array}{l} P_{A}^{h}=P_{A}^{c}=0.2\\ P_{B}^{h}=P_{B}^{c}\left(1-P_{A}^{h}\right)=0.2\left(1-0.2\right)=0.16. \end{array}$$ (11)

**Fig 2:**
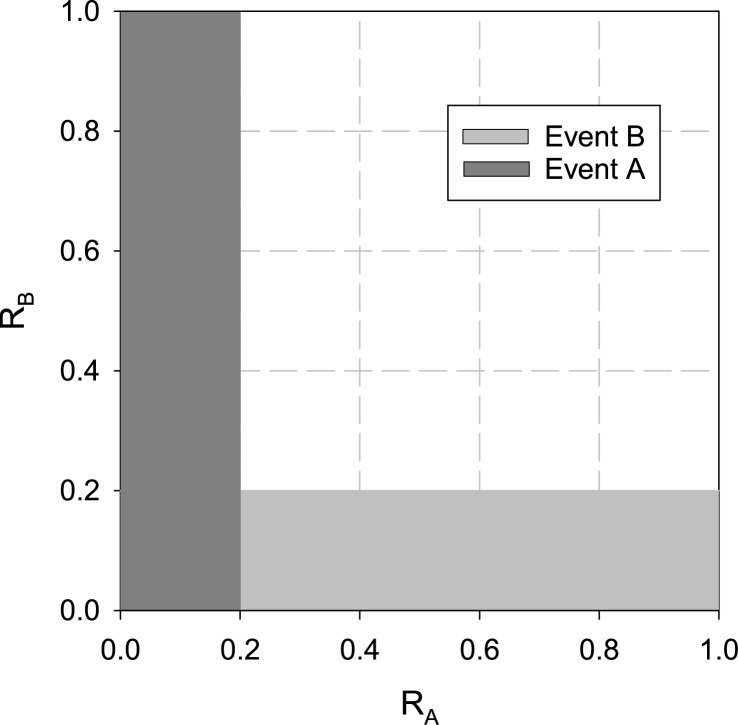
Example random process in which a pair of independent uniform numbers trigger event A if *R*_*A*_ < 0.2 and event B if *R*_*B*_ < 0.2. If *R*_*A*_ is observed before *R*_*B*_ and event A masks event B, then the hierarchical probability of observing event A is *P*^*h*^(*A*) = 0.2 and the hierarchical probability of observing event B is *P*^*h*^(*B*) = 0.16.

The hierarchical probabilities of events A and B are shown in Fig 2 as the shaded regions. Note from the figure that event A masks the joint probability of events A and B. The probability of neither events A nor B occurring for the hierarchical system is

$$P_{0}^{h}=1-P_{A}^{h}-P_{B}^{h}=1-0.2-0.16=0.64$$ (12)

which corresponds to the unshaded region in Fig 2.

Now, consider a naive model that simply returns the empirical, hierarchical probabilities of the non-events and the total events (binomial version) which may be broken down by A, B events (trinomial version). The same model will be applied in the binomial and trinomial versions to a test of 100 trials that also returns the expected results. That is, the test returns 64 non-events and 36 total events containing 20 A events and 16 B events. The *LL*_2_ for the binomial version of the model is

$$LL_{2}=64\;\ln(0.64)+36\;\ln(0.36)=-65.34.$$ (13)

The trinomial log likelihood is

$$LL_{3}=64\;\ln(0.64)+20\;\ln(0.20)+16\;\ln(0.16)=-90.07$$ (14)

whereas the deflated version of the trinomial log likelihood is

$$LL_{32}=64\;\ln(0.64)+(20+16)\;\ln(0.20+0.16)=-65.34.$$ (15)

We see that the deflated trinomial log likelihood is equivalent to the binomial log likelihood. This is as anticipated, since the same model was applied in binomial and trinomial versions to the same data set, so we would not expect one version of the model to be better than the other. In the results section below, we will use the deflated log likelihood in a standard *χ*^2^ test (see Eq (20)) to investigate whether adding the additional parameters to our trinomial DCS model (Eq (3)) improves the overall model fit when compared to the binomial model.

### Exact model gain and scaling parameters

In fitting probabilistic DCS models to empirical dive data, optimizing the adjustable parameters to maximize the log likelihood can be a compute-intensive and time-consuming task. Often, many initial parameter sets must be used in multiple optimizations to have some degree of confidence that the best values of the parameters are found. One of the difficulties that can arise in optimizing a model is that nearly-collinear parameters make the Hessian matrix ill-conditioned, resulting in slow convergence, poor accuracy, and creating other numerical problems [16, 25]. This is especially true of the model gain vector, $\vec{g}$, which has near collinearity with the tissue time parameters. This is also true of the near collinearity between the trinomal scale parameter, *a*, the gain vector, and the tissue time constants. In our previous work, we were able to find exact, although nonlinear and implicit, solutions for the optimal gain values for a simpler binomial problem [27]. The resulting exact solution gives the scientist the capability to remove the gain parameters from the optimization parameter space, which greatly improves the speed of optimization and the solution quality. This method for finding the exact gains can also be applied to the trinomial problem. In writing the exact gain and scale parameter equations, we will extend the shorthand notation of Eqs (4) and (5) and include time-of-symptom onset [17] in the formulation of exact gains. An overscript notation will now be used to show the integration interval for the hazard function as

$$\begin{array}{l} e^{-\vec{g}•\overset{01}{\vec{R}}}=e^{-\vec{g}•\int_{0}^{T1}\vec{r}dt}=\overset{01}{\xi }\\ e^{-\vec{g}•\overset{12}{\vec{R}}}=e^{-\vec{g}•\int_{T1}^{T2}\vec{r}dt}=\overset{12}{\xi }\\ e^{-\vec{g}•\overset{03}{\vec{R}}}=e^{-\vec{g}•\int_{0}^{\infty }\vec{r}dt}=\overset{03}{\xi }\\ e^{-\vec{g}•\overset{02}{\vec{R}}}=e^{-\vec{g}•\int_{0}^{T1}\vec{r}dt}e^{-\vec{g}•\int_{T1}^{T2}\vec{r}dt}=e^{-\vec{g}•\int_{0}^{T2}\vec{r}dt}=\overset{01}{\xi }\;\overset{12}{\xi }=\overset{02}{\xi } \end{array}$$ (16)

where the scale parameter of Eq (5) still applies and the integration limits *T*_1_ and *T*_2_ were previously discussed. Now, let a dive data set contain *Z* zero (non) events, *D* cases of DCS (binary model), *M* cases of mild DCS (trinomial model), and *S* cases of serious DCS (trinomial model) counted by the respective indices *z*,*d*,*m*, and *s*. Also, let a DCS model consist of *C* parallel tissue compartments. For the binary problem, the *c*^*th*^ optimal component, *g*_*c*_, of the gain vector is given by the simultaneous solution of the *C* equations

$$\sum_{d=1}^{D}\left[\frac{\overset{12}{R_{cd}}}{\overset{12}{\xi^{-1}}-1}-\overset{01}{R_{cd}}\right]=\sum_{z=1}^{Z}\overset{03}{R_{cz}}$$ (17)

where *R*_*cp*_ denotes the integrated hazard function for the *c*^*th*^ tissue compartment on the *p*^*th*^ dive profile of the data subset (*D* or *Z*). The optimal *c*^*th*^ compartmental gain, *g*_*c*_, for the trinomial DCS model is given by the simultaneous solution of the *C* equations

$$\sum_{s=1}^{S}\left[\frac{a{\overset{12}{R}}_{cs}}{\overset{12}{\xi^{-a}}-1}-\left(1+a\right){\overset{01}{R}}_{cs}\right]+\sum_{m=1}^{M}\left[\frac{{\overset{12}{R}}_{cm}}{\overset{12}{\xi^{-1}}-1}-{\overset{01}{R}}_{cm}-a{\overset{02}{R}}_{cm}\right]=\left(a+1\right)\sum_{z=1}^{Z}{\overset{03}{R}}_{cz}$$ (18)

together with the solution of the equation for the optimal scale parameter, *a*

$$\sum_{s=1}^{S}\left[\;\ln\left(\overset{01}{\xi_{s}}\right)-\frac{\;\ln\left(\overset{12}{\xi_{s}}\right)}{\overset{12}{\xi_{s}^{-a}}-1}\right]=-\sum_{m=1}^{M}\;\ln\left(\overset{02}{\xi_{m}}\right)-\sum_{z=1}^{Z}\;\ln\left(\overset{03}{\xi_{z}}\right).$$ (19)

In finding the optimal gain components for the binary problem (solutions of Eq (17)) or the optimal gain components and scale parameter for the trinomial problem (solutions of Eqs (18) and (19)), the integrated hazard functions, *R*, are constant and need only be evaluated once for each optimal gain set. A proof of gain vector optimality for the binomial model may be found in our previous work [27] while a similar proof for the trinomial model is presented in the Appendix.

### DCS model optimization system and statistical methods

Our previously-developed probabilistic DCS optimization and modeling system [25] was used to generate the results for this work. We extended the previous capabilities of this system by adding the trinomial model and by adding the exact gain and scale parameter solutions. For calculation of 95% confidence limits on model parameters, we used our software and standard calculation methods [28]. To assign 95% confidence limits and 95% prediction limits on model fits to the data, we used SigmaPlot v11 [29]. For a test of model improvement, we used the log likelihood difference test [28]. A value of *p* < 0.05 was considered significant and a value of *p* < 0.01 was considered highly significant.

## Results

In the results subsections to follow, an examination will be made of the changes in model fit and prediction between the trinomial and binomial models. We will then compare the binomial model to the trinomial model. The comparison will include parameter values with confidence intervals, changes in model prediction vs. observed DCS incidences, a study of DCS event probability shifts between the models, and DCS cumulative density functions.

### Trinomial model vs. binomial model

We will first examine the change in model fit between the binomial and trinomial models using DCS severity defined as Types A and B (Table 1). In order to do this, the log likelihood difference test [28] will be used together with our deflated log likelihood (Eq (10)). The model parameter values, 95% confidence intervals on the parameter values, and log likelihood values are shown for all models in Table 2. In comparing the binomial and trinomial models, we can directly compare *LL*_2_ with *LL*_32_ as shown in the methods section. This comparison results in the log likelihood difference value

$$\Delta LL=\chi^{2}=-2\left[LL_{2}-LL_{32}\right]=182.28$$ (20)

whereas the critical *χ*^2^ value for significance (*p* < 0.05) for the single additional degree of freedom added to the binomial model to generate our trinomial model is 3.841 and the critical *χ*^2^ for high significance (*p* < 0.01) is 6.635. Therefore, the trinomial model offers a highly significant improvement over the binomial model (*p* ≪ 0.01). This highly significant improvement in model performance of the trinomial system over the binomial system may be understood by examining the empirical event probabilities. There is a far lower incidence of serious DCS (20/3322) than mild DCS (170/3322) in the BIG292 data set. The trinomial model predicts a lower probability of occurrence for these serious events as well as for the mild events than does the binomial model. The lowered probability increases the deflated log likelihood, thus improving the model fit.

**Table 2:** Parameter values and 95% confidence intervals for the binomial and trinomial (Type A/B classification) variants of the LE1nt model. For the log likelihood values, the underlined results can be directly compared. The trinomial model offers a highly significant improvement over the binomial model.

	*Binomial*	*Trinomial*
k_1_ (min)	1.593 ± 0.280	1.593 ± 0.284
k_2_ (min)	184.1 ± 21.37	184.7 ± 21.84
k_3_ (min)	535.7 ± 94.14	535.5 ± 94.36
g_1_ (min^-1^)	3.290E-3 ± 1.275E-3	2.947E-3 ± 1.150E-3
g_2_ (min^-1^)	3.908E-4 ± 9.733E-5	3.508E-4 ± 8.959E-5
g_3_ (min^-1^)	2.727E-4 ± 9.767E-5	2.443E-4 ± 8.808E-5
PXO_1_ (fsw)	∞	∞
PXO_2_ (fsw)	0.259 ± 0.054	0.260 ± 0.064
PXO_3_ (fsw)	∞	∞
a	-	0.116 ± 0.0275
LL_2_	-978.76	-
LL_3_	-	-1042.66
LL_32_	-	-887.62

The model parameters for all models are also shown in Table 2. The compartmental tissue time constants, *k*_1–3_, are nearly identical between the optimized binomial and optimized trinomial models. As a result, the compartmental gain parameters, *g*_1–3_, are driven to smaller values by this optimization process because there are fewer events in the mild DCS category with the trinomial model. This may be understood by realizing that the probabilities of each event type, mild DCS and serious DCS, must both diminish when the separate events are not lumped into a single event category.

**Fig 3:**
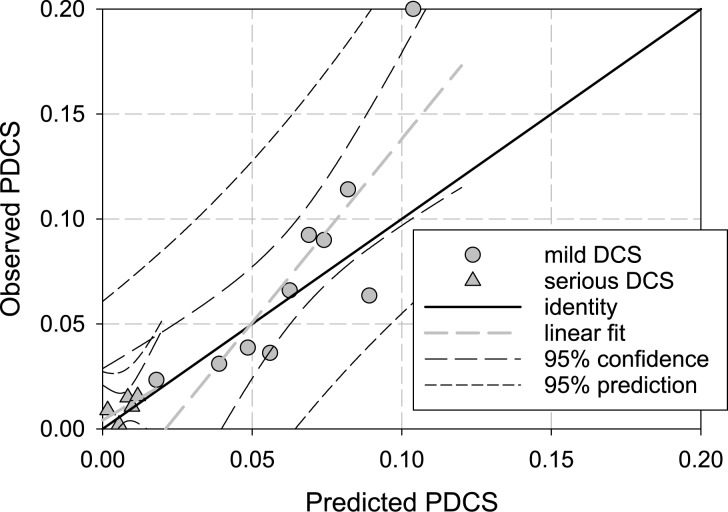
Trinomial model predicted probability of DCS versus observed probability of DCS. These points were generated by sorting the mild (serious) profiles into ten (five) bins, each bin containing the same number of observed DCS cases. The predicted and observed probabilities of DCS were then calculated for each bin. The data are shown together with a linear fit (gray, long dash $r_{mild}^{2}=0.69,\;r_{serious}^{2}=0.37$) and the 95% confidence (black, long dash) and 95% prediction (black, short dash) bands.

In Fig 3, the predicted probability of DCS is plotted against the observed probability of DCS for the trinomial model. These results were generated by sorting the entire dive data set by predicted DCS probability into ten (mild) or five (serious) bins with each bin containing an equal number of observed DCS events. The data points shown in Fig 3 are the number of serious DCS cases (triangles) and mild DCS cases (circles), expressed as a probability, for all of the dive profiles in that bin. Also shown in the figure is the line of identity. For a perfect model, all data points would fall on the line of identity. The gray dashed lines correspond to linear fits of the observed DCS to predicted DCS ($r_{mild}^{2}=0.69,\;r_{serious}^{2}=0.37$). The 95% confidence bands are shown by long-dashed lines while the 95% prediction bands are shown by short-dashed lines. As indicated by the figure, the trinomial version of the LE1nt model reasonably predicts both the serious and mild DCS trends but there remains considerable opportunity for improvement in the predictive performance of the underlying DCS model. Further modeling improvements could include the use of mixed pharmacokinetic models and more physiologically-based models. Certainly, additional human dive trials would be helpful. One of the issues that we (and others) are currently attempting to address is how to best model heterogenous data sets such as BIG292 that include such diverse dives as 600 fsw dives lasting less than one minute with saturation dives; for example, 20 fsw dives lasting more than 10,000 minutes.

### Predictions on data

In Table 3, we show the number of observed DCS cases along with the number of predicted DCS cases for each sub-set that makes up the entire BIG292 data set. The data consist of single air, repetitive and multilevel air, single non-air, repetitive and multilevel non-air, and saturation dives. The data show the number of mild and serious DCS cases contained in each data set. Note that the total does not include any fractionally-weighted marginal cases. That is, only mild and serious cases are included in the total. For each data set, we also show the number of cases as predicted by the binomial and trinomial models. The 95% confidence band in the total number of predicted cases in the BIG292 dataset is also shown in this table. In comparing the predictions of the binomial to the trinomial model, we see that the number of predicted cases for the binomial model is equal to the number of mild plus serious cases for the trinomial model.

**Table 3:** DCS occurrences and binomial and trinomial model (Type A/B classification) predictions for the BIG292 data set.

					Predicted DCS		
		Observed DCS			Binomial	Trinomial	
	Exposures	Mild	Serious	Total	Total	Mild	Serious
**Single Air**							
EDU885A	483	27	3	*30*	25.6	22.9	2.7
DC4W	244	7	1	*8*	5.4	4.9	0.6
SUBX87	58	0	2	*2*	0.6	0.5	0.1
NMRNSW	91	4	1	*5*	6.2	5.5	0.7
PASA	72	4	1	*5*	3.1	2.8	0.3
NSM6HR	57	3	0	*3*	4.7	4.2	0.5
**Repet & Multilevel Air**							
EDU885AR	182	11	0	*11*	10.8	9.7	1.2
DC4WR	12	3	0	*3*	0.8	0.7	0.1
PARA	135	6	1	*7*	9.8	8.7	1.1
PAMLA	236	9	4	*13*	19.2	17.1	2.1
**Single Non Air**							
NMR8697	477	9	2	*11*	18.5	16.5	2.0
EDU885M	81	4	0	*4*	3.4	3.0	0.4
EDU1180S	120	9	1	*10*	7.5	6.7	0.8
**Repet & Multilevel Non Air**							
EDU184	239	11	0	*11*	14.3	12.7	1.5
PAMLOAD	134	5	1	*6*	8.0	7.2	0.9
PAMLOAS	140	5	0	*5*	7.1	6.3	0.8
EDU885S	94	4	0	*4*	4.6	4.2	0.5
**Saturation**							
ASATEDU	120	11	2	*13*	10.4	9.3	1.1
ASATNMR	50	1	0	*1*	3.6	3.2	0.4
ASATNSM	132	18	0	*18*	14.2	12.7	1.6
ASATARE	165	19	1	*20*	12.6	11.2	1.4
*Total*	*3322*	*170*	*20*	*190*	*190*.*4* ± *28*.*4*	*169*.*9* ± *23*.*6*	*20*.*5* ± *8*.*9*

### Binomial to trinomial probability shift

**Fig 4:**
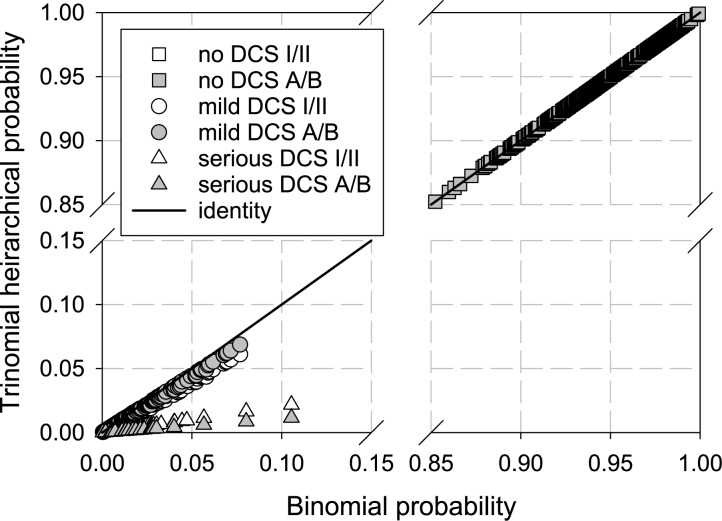
Binomial to trinomial probability shift plot for the I/II (unfilled symbols) and A/B (filled symbols) predictions. For the dive profiles that result in DCS, the binomial predicted probability is compared to the predicted DCS probability from the trinomial model. Profiles resulting in serious DCS are shown by triangles and profiles resulting in mild DCS are shown by circles. For dive profiles that did not produce DCS, the probability of no DCS is shown by squares. If a point falls below the line of identity, the trinomial model predicts a lower probability of the corresponding event on that dive profile. The probabilities of mild and serious DCS under the trinomial model with either I/II or A/B classifications are smaller than the probability of DCS under the binomial model while the probability of no DCS is nearly the same for both models with either classification. Note that the A/B no DCS outcomes obscure the I/II no DCS outcomes.

The shift in the predicted probabilities of the DCS events between the binomial and trinomial models is shown in Fig 4 for both the I/II and A/B classifications of the events. This shift-plot shows the probabilities of no DCS, mild DCS, and serious DCS as calculated by the binomial and trinomial models along with the line of identity. The probability of DCS under the binomial model is plotted on the abscissa and the probability of DCS or no DCS under the trinomial model for that same dive profile is plotted on the ordinate. Thus, a point that falls below the line of identity has a lower probability of the predicted event under the trinomial model than under the binomial model. We show the model-predicted probability of both mild and serious DCS in the lower left region of the plot using both the I/II and A/B classifications. In the upper right region of this same plot, we show the probability of DCS not occurring. It is worth noting that the trinomial model negligibly affects the prediction of no-DCS. This is indicated on the plot by the fact that the no-DCS predictions fall so closely to the line of identity. Additionally, the no DCS events shown in Fig 4 fall on top of one another so the A/B no DCS profiles obscure the I/II DCS profiles. As the figure shows, both the mild and serious DCS event types have a lower predicted probability under the trinomial model than under the binomial model, the I/II mild DCS probabilities are somewhat less than the A/B mild DCS probabilities, and the I/II serious probabilities are somewhat greater than the A/B serious probabilities. These observations are consistent with what should be expected from the trinomial grading of the dive data and an examination of the data bulk probabilities.

Another feature of the predicted probabilities, shown in Fig 4, is the tight correlation of the predicted probabilities as indicated by the lack of scatter about a straight line that could be drawn through the points for each event type. This could be anticipated by the fact that the optimized model parameters, reported in Table 2, are nearly identical for both the binomial and trinomial models. If we fit a straight line to the mild and serious event predicted probability points shown in the figure, the straight lines have the respective slopes of 0.894 (*r*^2^ > 0.999) and 0.109 (*r*^2^ > 0.999). The slopes are useful as a basic “rule of thumb” for understanding the probability reduction in going from the binomial to the trinomial model. For example, given a predicted probability of binomial DCS, *P*_2_, the probabilities of trinomial mild DCS and trinomial serious DCS are *P*_m_ ≈ 0.894*P*_2_ and *P*_s_ ≈ 0.109*P*_2_ respectively.

**Fig 5:**
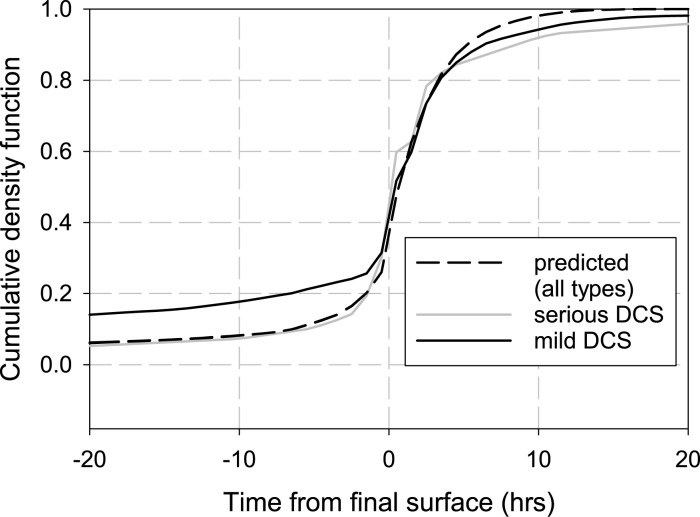
Trinomial cumulative density function for the serious DCS cases (grey, solid curve) and mild DCS cases (black, solid curve). The cumulative density function for predicted mild and serious DCS fall on the same curve (black, dashed curve).

The cumulative density function (CDF) is a useful tool that allows for comparison of DCS symptom onset times. Due to the fact that our present hierarchical models simply scale the event probabilities and do not allow the dynamics of DCS to vary from one severity category to the next, the CDF shown in Fig 5 indicates that all model predictions collapse onto the same curve (dashed black line). This contrasts with the observed mild (solid black line) and serious (solid gray line) DCS cumulative probabilities, which are shown to be different from approximately 15 to 3 hours before surfacing and also from approximately 3 hours after surfacing onward. In both cases, the observed cumulative probability of serious DCS is less than that of mild DCS. The model does the best job of predicting cumulative probability from the time of surfacing to approximately 3 hours after surfacing. Before surfacing, the model is seen to significantly under-predict the cumulative probability of DCS; whereas after approximately 3 hours after surfacing, the model is seen to over-predict the cumulative probability of DCS. In other words, the model predicts that more of the DCS events of all types occur after surfacing than the data indicates.

### Series expansion on the probability of mild DCS

After the model optimization is complete and the scale parameter is found, it is no longer necessary to separately evaluate a dive profile for the hierarchical probabilities of the differing event types. With the optimized scale parameter, *a*, the hierarchical probability of serious DCS may be calculated directly from the hierarchical probability of mild DCS. To demonstrate this, we begin with the hierarchical probabilities of serious and mild DCS written in terms of Eqs (4) and (5) as

$$P_{s}=1-\xi^{a},\;P_{m}=\xi^{a}-\xi^{a+1}.$$ (21)

Next, we can eliminate *ξ* between the two Eq (21) to get

$$P_{m}=\left(1-P_{s}\right)-{\left(1-P_{s}\right)}^{1+\frac{1}{a}}.$$ (22)

While this is a useful equation, a second order series expansion of Eq (22) for small event probabilities provides a more insightful result. The series expansion is

$$P_{m}=\frac{1}{a}P_{s}-\frac{a+1}{2a^{2}}P_{s}^{2}+O\left(P_{s}^{3}\right).$$ (23)

The first term on the right hand side of Eq (23) is a simple scaling of the event probability while the second term reduces the probability of mild DCS with increasing probability of serious DCS due to the hierarchical connection between the two event types. A more useful version of Eq (23) is

$$P_{s}=\frac{a}{a+1}\left(1-\sqrt{1-2P_{m}\left(a+1\right)}\right)\;\forall \;P_{m}<0.18$$ (24)

which expresses the lower probability of serious DCS as a function of the greater mild DCS probability. Eq (24) is accurate to within 1% of the probability as calculated by Eq (6) (first line) for dive profiles whose probability of mild DCS is less than approximately 0.18. Given the DCS probabilities displayed in Fig 4, Eq (24) is appropriate for all dives in the BIG292 dataset. It is important to note that Eqs (22–24) cannot be used without first optimizing the model parameters with the trinomial DCS model.

## Discussion

From the log likelihood comparisons between the binomial and trinomial models, we can conclude that there is a highly significant improvement in the fit made by the simple hierarchical scaling we develop in this paper. We will now return to an observation made earlier in this paper that the current U.S. Navy guidance for acceptable risk of DCS occurrence for any given dive is 2% for ‘mild’ DCS and 0.1% for ‘serious’ DCS, a 20:1 ratio. This contrasts with the prevalence of ‘mild’ and ‘serious’ DCS events in the BIG292 dataset as summarized in Table 1. To elaborate, the Type I/II definition of severity gives 152 ‘mild’ DCS events and 38 ‘serious’ DCS events for a 4:1 ratio while the Type A/B definition of severity gives 170 ‘mild’ events and 20 ‘serious’ DCS events for an 8.5:1 ratio. The disparity between the 20:1 ratio stated in U.S. Navy guidance and the 4:1 or 8.5:1 ratios in the dive data has implications for establishing dive procedures according to ‘mild’ and ‘serious’ DCS.

**Fig 6:**
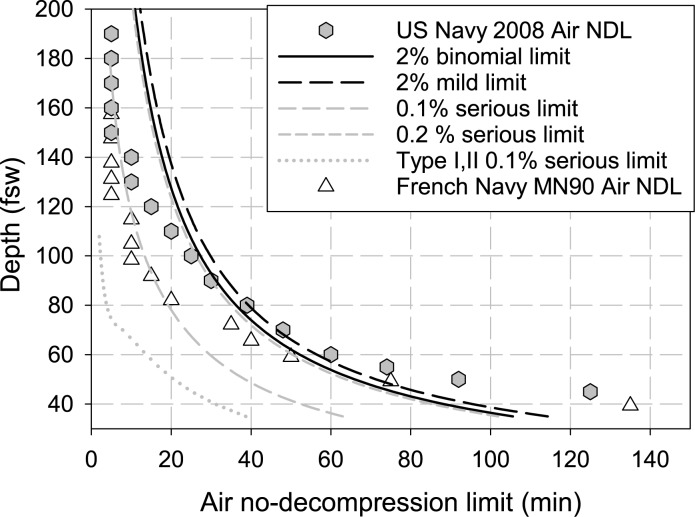
Air no-decompression limit and model predictions. The filled hexagonal symbols indicate the bottom depth in feet of seawater (fws) versus the air no-decompression limit in minutes according to the 2008 US Navy Diving Manual (rev 6). The unfilled triangles indicate the air no-decompression limits according to the French Navy NM90 tables. The solid black curve shows the 2% limit as predicted by the binomial LE1nt model. The black dashed curve is the corresponding 2% limit for mild DCS as predicted by the trinomial model. The medium dashed grey curve shows the 0.1% limit for serious DCS as predicted by the trinomial LE1nt model, while the short dashed grey curve shows the operationally consistent 0.2% limit for serious DCS as predicted by the trinomial LE1nt model. The grey dotted curve shows the 0.1% serious limit as predicted by using the Type I/II definition. Note that the predicted 0.1% serious DCS limit is well below the established U.S. Navy air no-decompression limit but corresponds well with the French NM90 limit in the range of 80–120 fsw.

We can demonstrate these implications by considering the air no-decompression limits (NDL) in the USN 2008 Dive Manual [30]. In Fig 6, we show the published U.S. Navy air no-decompression limits as a function of depth using filled hexagons. For comparison, we also show the French Navy NM90 table air no-decompression limit using unfilled triangles. Also shown on this plot, by the solid black line, is the 2% DCS probability line for the binomial model as predicted by the LE1nt model using the optimized parameters listed in Table 2. Note that the 2% binomial limit over-predicts the available bottom time for the deeper dives and under-predicts the available bottom time for the shallower dives when compared to the NDL. If we plot the output from the trinomial model for the Type I/II definition of DCS severity, we find that the reduced probability of predicted ‘mild’ DCS results in a longer allowable bottom time for the same 2% probability limit. This is indicated by the black curve in Fig 6. However, when we plot the 0.1% limit for ‘serious’ DCS, we find a vastly decreased allowable bottom time for all depths. Interestingly, the French NM90 air no-decompression times correspond well with the 0.1% serious DCS limit in the range 80–120 fsw and result in a relatively low incidence of serious DCS [31]. The take-home message is that if a given dive must be within acceptable risk limits for both ‘mild’ (2%) and ‘serious’ (0.1%) DCS in accordance with the previous analysis, the NDL exposures will be restricted to the limits for ‘serious’ DCS.The previous analysis identified Type II DCS as ‘serious’ and Type I DCS as ‘mild’ with a 4:1 ratio of Type I to Type II cases. Since some Type II cases only have subjective manifestations (Table 1), however, defining Type II as ‘serious’ does not appear to meet the U.S. Navy definition of acceptable ‘serious’ DCS as discussed in the Introduction. Type B DCS, which does not include subjective manifestations, may be a more reasonable definition of ‘serious’ DCS (Table 1) with a ratio of Type A to Type B cases of 8.5:1 rather than 4:1 as for Type I and II. One must be certain that ‘serious’ DCS cases in the calibration data are truly ‘serious’ to avoid overly restrictive diving limits as in Fig 6. While Type B DCS meets this requirement better than Type II DCS, the case descriptions provided by Temple [5, 6], from which Table 1 was derived, do not provide sufficient information about therapy that might reject some of the 20 Type B cases as ‘mild’ rather than ‘serious.’ Differentiation of ‘mild’ from ‘serious’ DCS is a challenge that we discuss elsewhere [15]. Only when the A/B severity scoring is used and the acceptable limit of serous DCS is increased from 0.1% to 0.2% is the trinomial model operationally consistent with the air NDLs. This is shown in Fig 6 by the near correspondence of the 2.0% mild DCS limiting curve (black dashed line) and the 0.2% serious limiting curve (gray short dashed curve) and the reasonable agreement of these curves with the U.S. Navy no decompression limits.The practical importance of inaccurate identification of ‘serious’ cases can be illustrated by comparing the 60 fsw NDL bottom times for ‘mild’ and ‘serious’ DCS based on Type I/II (Fig 6) as opposed to Type A/B cases. For Type I/II, the dive times are 61 min for the 2% ‘mild’ limit and 14 min for the 0.1% ‘serious’ limit. In contrast, Type A/B requires dive times of 56 min for the 2% limit and 30 min for the 0.1% limit. Knowledge of the true ratio of ‘mild’ to ‘serious’ cases is essential for application of different acceptable DCS limits according to severity. This will require a larger dataset than BIG292 that we used here and more complete DCS case descriptions to facilitate accurate selection of ‘serious’ cases.

It is worthwhile pointing out the possibility that the “PFO effect” might lead to an increased risk of serious DCS. While U.S. Navy divers are not screened for a patent foramen ovale (right-to-left shunt), one case control study found and increased presence of PFOs in civilian divers treated for DCS [32]. The study, which screened 101 divers treated for DCS and had a control group of 101 healthy divers, found that the prevalence of PFO in the group undergoing treatment was 58.4% while the prevalence was 24.8% in the control group (odds ratio, 4.3; p = 0.09). The investigators concluded that an increased incidence of cerebral and cochleovestibular manifestations were associated with major right-to-left shunting. Another avenue for further research relates to the different T_1_ and T_2_ times that were observed for ‘mild’ and ‘serious’ DCS (Fig 5). In developing our hierarchical DCS models we added a simple scale parameter to distinguish between events of differing severity and, in this work, did not add any adjustable parameters that would allow the dynamics of the various event types to change. This last point is made clear by examining the occurrence density functions (ODF) for the trinomial model. The ODF curves for this model show that the predicted event onset occurring at the same time regardless of the event severity. We expect that adding one or more free parameters that could be adjusted during the fitting process would result in better predictive capability of the DCS models. Even when this is accomplished, we will not be able to escape the underlying empirical probabilities of mild and serious DCS which give us a 4:1 ratio of the event types—at least for the BIG292 dataset.

## Conclusion

In this work, we have presented a rigorous approach for simultaneously predicting multinomial outcomes from a single probabilistic DCS model. In developing the trinomial (3 state–no DCS, mild DCS, serious DCS) model, we demonstrated the connection between hierarchical (observed) and competitive (predicted) probabilities. Using these hierarchical and competitive probabilities, we developed the binomial and trinomial log likelihood functions. Further, we were able to derive a deflated likelihood that collapses the trinomial log likelihood onto a 2-state equivalent which allowed for a direct log likelihood comparison between the binomial and trinomial models.

The retrospectively-categorized data used for model calibration and analysis contained 3322 air and N_2_-O_2_ dives covering depth ranges of 20 to 602.4 feet of seawater and durations of 0.4 to 12,960 minutes resulting in 190 cases of DCS and 110 marginal DCS cases. Following our previous work, we scored the marginal DCS cases as non-events. For the trinomial model, we considered two different scorings of mild and serious DCS. The first of these two scorings, Type I/II, considered mild DCS (type I) as composed of pain, lymphatic or skin, and constitutional or nonspecific manifestations while serious DCS (type II) as composed of serious neurological, cardiopulmonary, and mild neurological manifestations. This Type I/II scoring was consistent with current U.S. Navy definitions of mild and serious DCS. The second type of severity scoring, Type A/B, considered mild DCS (type A) as composed of mild neurological, pain, lymphatic or skin, and constitutional or nonspecific manifestations while serious DCS (type B) as composed of serious neurological, and cardiopulmonary manifestations.

Using a previously-developed and validated DCS model, the LE1nt model, we optimized the model parameters for the 2-state binomial and 3-state trinomial models using both the Type I/II and A/B data scoring. We used the deflated log likelihood to demonstrate that the trinomial model offers a highly significant improvement in model fit to the data when compared to the binomial model. The hierarchical framework we developed in this paper is not specific to the particular DCS model we used for this work but is general and may be used with any of the current U.S. Navy operational and experimental DCS models as well as many of the recently-published probabilistic models from the scientific and medical literature.

Using U.S. Navy guidance of a 2.0% acceptable risk of mild DCS and a 0.1% acceptable risk of serious DCS, we explored the binomial and trinomial model predictions for the current U.S. Navy air no-decompression limits. For the binomial model, the air NDLs are inside the 2.0% DCS limit (incidence only) for depths greater than approximately 80 fsw and slightly outside of this limit for shallower depths. For the trinomial model with the I/II scoring of DCS severity, the 0.1% serious limit controls the allowable bottom time and would severely restrict current NDLs. With the A/B scoring of DCS severity, the 0.1% serious limit still set the allowable bottom time but did not limit the bottom time as severely as did the I/II scoring. The trinomial model only agreed with the U.S. Navy air no decompression limits when the A/B severity scoring was used and the acceptable risk of serious DCS was increased to 0.2%. We conclude by strongly cautioning that the results presented in this paper should not be used for dive planning until this work has been rigorously validated by human dive trials.

## Appendix

*Claim*: The optimal tissue compartment gains, *g*_*c*_, and scale parameter, *a*, for the trinomial model using DCS symptom onset times are given by the simultaneous solution of the equations

$$\sum_{s=1}^{S}\left[\frac{a{\overset{12}{R}}_{cs}}{\overset{12}{\xi^{-a}}-1}-\left(1+a\right){\overset{01}{R}}_{cs}\right]+\sum_{m=1}^{M}\left[\frac{{\overset{12}{R}}_{cm}}{\overset{12}{\xi^{-1}}-1}-{\overset{01}{R}}_{cm}-a{\overset{02}{R}}_{cm}\right]=\left(a+1\right)\sum_{z=1}^{Z}{\overset{03}{R}}_{cz}$$ (25)

and

$$\sum_{s=1}^{S}\left[\;\ln\left(\overset{01}{\xi_{s}}\right)-\frac{\;\ln\left(\overset{12}{\xi_{s}}\right)}{\overset{12}{\xi_{s}^{-a}}-1}\right]=-\sum_{m=1}^{M}\;\ln\left(\overset{02}{\xi_{m}}\right)-\sum_{z=1}^{Z}\;\ln\left(\overset{03}{\xi_{z}}\right).$$ (26)

*Proof*: Let a trinomial decompression model be described by the hierarchical probability functions listed in Eq (6). Let the trinomial log likelihood be given as Eq (9). Further, let the time of onset be described by the risk function integrals as specified in Eq (16). Additionally, let a dive data set consist of the respective *Z* profiles that did not result in DCS, *M* profiles that resulted in mild DCS, and *S* profiles that resulted in serious DCS. Let these dive profiles be counted by the respective indices *z*, *m*, and *s*. Begin by substituting the definitions for mild, serious, and no DCS for the hierarchical trinomial probabilities into the trinomial log likelihood function. This results in the equation

$$LL_{3}=\sum_{s=1}^{S}\;\ln\left(\overset{01}{\xi_{s}^{a+1}}-{\overset{01}{\xi }}_{s}\overset{02}{\xi_{s}^{a}}\right)+\sum_{m=1}^{M}\;\ln\left(\overset{01}{\xi_{m}}\overset{02}{\xi_{m}^{a}}-\overset{02}{\xi_{m}^{a+1}}\right)-\left(a+1\right)\sum_{z=1}^{Z}\;\ln\left({\overset{03}{\xi }}_{z}\right).$$ (27)

To find the optimal gain, we take the derivative of *LL*_3_ with respect to (w.r.t.) *g*_*c*_ to write the equation

$$\frac{\partial }{\partial g_{c}}\left(LL_{3}\right)=\sum_{s=1}^{S}\frac{({\overset{01}{R}}_{cs}+a{\overset{02}{R}}_{cs})\overset{01}{\xi_{s}}\overset{02}{\xi_{s}^{a}}-(a+1){\overset{01}{R}}_{cs}\overset{01}{\xi_{s}^{a+1}}}{\overset{01}{\xi_{s}^{a+1}}-\overset{01}{\xi_{s}}\overset{02}{\xi_{s}^{a}}}+\sum_{m=1}^{M}\frac{(a+1){\overset{02}{R}}_{cm}\overset{02}{\xi_{m}^{a+1}}-(\overset{01}{R_{cm}}+a\overset{02}{R_{cm}})\overset{01}{\xi_{m}}\overset{02}{\xi_{m}^{a}}}{{\overset{01}{\xi }}_{m}\overset{02}{\xi_{m}^{a}}-\overset{02}{\xi_{m}^{a+1}}}-(a+1)\sum_{z=1}^{Z}\overset{03}{R_{cz}}.$$ (28)

Similarly, the optimal scale parameter, *a*, may be found by taking the derivative of *LL*_3_ w.r.t parameter *a*; resulting in

$$\frac{\partial }{\partial a}\left(LL_{3}\right)=\sum_{s=1}^{S}\frac{\;\ln(\overset{02}{\xi })\overset{01}{\xi_{s}}\overset{02}{\xi_{s}^{a}}-\;\ln(\overset{01}{\xi_{s}})\overset{01}{\xi_{s}^{a+1}}}{\overset{01}{\xi_{s}^{a+1}}-\overset{01}{\xi_{s}}\overset{02}{\xi_{s}^{a}}}+\sum_{m=1}^{M}\frac{\;\ln(\overset{02}{\xi_{m}})\overset{02}{\xi_{m}^{a+1}}-\;\ln(\overset{02}{\xi_{m}})\overset{01}{\xi_{m}}\overset{02}{\xi_{m}^{a}}}{\overset{01}{\xi_{m}}\overset{02}{\xi_{m}^{a}}-\overset{02}{\xi_{m}^{a+1}}}-\sum_{z=1}^{Z}\;\ln(\overset{03}{\xi_{z}}).$$ (29)

Next, eliminate $\overset{01}{\xi_{s}^{a+1}}=\overset{01}{\xi_{s}}\overset{01}{\xi_{s}^{a}}$ from the first term and the product $\overset{01}{\xi_{m}}\overset{02}{\xi_{m}^{a}}$ from the second term of the right-hand-sides of Eqs (28) and (29) to produce

$$\frac{\partial }{\partial g_{c}}\left(LL_{3}\right)=\sum_{s=1}^{S}\frac{({\overset{01}{R}}_{cs}+a{\overset{02}{R}}_{cs})\overset{12}{\xi_{s}^{a}}-(a+1){\overset{01}{R}}_{cs}}{1-\overset{12}{\xi_{s}^{a}}}+\sum_{m=1}^{M}\frac{\left(a+1){\overset{02}{R}}_{cm}\overset{12}{\xi_{m}}-(\overset{01}{R_{cm}}+a\overset{02}{R_{cm}}\right)}{1-\overset{12}{\xi_{m}}}-(a+1)\sum_{z=1}^{Z}\overset{03}{R_{cz}}$$ (30)

and

$$\frac{\partial }{\partial a}\left(LL_{3}\right)=\sum_{s=1}^{S}\frac{\;\ln(\overset{02}{\xi })\overset{02}{\xi_{s}^{a}}-\;\ln(\overset{01}{\xi_{s}})\overset{01}{\xi_{s}^{a}}}{\overset{01}{\xi_{s}^{a}}-\overset{02}{\xi_{s}^{a}}}+\sum_{m=1}^{M}\frac{\;\ln(\overset{02}{\xi_{m}})\overset{02}{\xi_{m}}-\;\ln(\overset{02}{\xi_{m}})\overset{01}{\xi_{m}}}{\overset{01}{\xi_{m}}-\overset{02}{\xi_{m}}}-\sum_{z=1}^{Z}\;\ln(\overset{03}{\xi_{z}}).$$ (31)

Finally, we use the relationship $\overset{02}{R}=\overset{01}{R}+\overset{12}{R}$ to rewrite Eqs (30) and (31) as

$$\frac{\partial }{\partial g_{c}}\left(LL_{3}\right)=\sum_{s=1}^{S}\left[\frac{a{\overset{12}{R}}_{cs}}{\overset{12}{\xi_{s}^{-a}}-1}-\left(1+a\right)\overset{01}{R_{cs}}\right]+\sum_{m=1}^{M}\left[\frac{{\overset{12}{R}}_{cm}}{\overset{12}{\xi_{m}^{-1}}-1}-\overset{01}{R_{cm}}-a{\overset{02}{R}}_{cm}\right]-(a+1)\sum_{z=1}^{Z}\overset{03}{R_{cz}}$$ (32)

and

$$\frac{\partial }{\partial a}\left(LL_{3}\right)=\sum_{s=1}^{S}\left[\frac{\;\ln(\overset{12}{\xi })}{\overset{02}{\xi_{s}^{-a}}-1}-\;\ln(\overset{01}{\xi })\right]+\sum_{m=1}^{M}\;\ln(\overset{02}{\xi_{m}})-\sum_{z=1}^{Z}\;\ln(\overset{03}{\xi_{z}}).$$ (33)

We find the stationary gains and scale factor by equating Eqs (32) and (33) to zero to give Eqs (25) and (26) and solving these simultaneous equations.

The next step in the proof of optimality is to show that the second derivatives of *LL*_3_ w.r.t. *g*_*c*_ and *a* are negative so that we know the stationary point given by the simultaneous solutions of the *C* Eq (32) together with the solution of Eq (33) corresponds to a maximum. These second derivatives are

$$\frac{\partial^{2}}{\partial g_{c}^{2}}\left(LL_{3}\right)=-\sum_{s=1}^{S}\frac{a^{2}\overset{12}{R_{cs}^{2}}\overset{12}{\xi^{-a}}}{{\left(\overset{12}{\xi^{-a}}-1\right)}^{2}}-\sum_{m=1}^{M}\frac{\overset{12}{R_{cm}^{2}}\overset{12}{\xi }}{{\left(\overset{12}{\xi^{-1}}-1\right)}^{2}}$$ (34)

and

$$\frac{\partial^{2}}{\partial a^{2}}\left(LL_{3}\right)=-\sum_{s=1}^{S}\frac{\overset{12}{\xi^{-a}}\;\ln\left({\left(\overset{12}{\xi }\right)}^{2}\right)}{{\left(\overset{12}{\xi^{-a}}-1\right)}^{2}}.$$ (35)

Since the quantities *a*, *R*, and *ξ* are nonnegative, inspection of Eqs (34) and (35) reveals that the second derivatives are negative and the stationary point given by the simultaneous solution of Eqs (32) and (33) is a maximum.

QED.
